# Validation of a Real-Time PCR for the Detection of *Mycobacterium tuberculosis* Complex Members in Bovine Tissue Samples

**DOI:** 10.3389/fvets.2019.00061

**Published:** 2019-03-04

**Authors:** Victor Lorente-Leal, Emmanouil Liandris, Elena Castellanos, Javier Bezos, Lucas Domínguez, Lucía de Juan, Beatriz Romero

**Affiliations:** ^1^VISAVET Health Surveillance Center, Complutense University of Madrid, Madrid, Spain; ^2^Animal Health Department, Veterinary Faculty, Complutense University of Madrid, Madrid, Spain; ^3^Exosome Diagnostics Inc., Waltham, MA, United States

**Keywords:** real-time PCR, *Mycobacterium tuberculosis* complex, tuberculosis, detection, bovine tissue

## Abstract

Although the post-mortem diagnosis of bovine tuberculosis is mainly achieved through microbiological culture, the development of other techniques to detect *Mycobacterium tuberculosis* complex (MTBC) members directly from tissue samples has been pursued. The present study describes the development, optimization and validation of a Real-Time PCR based on the *mpb70* gene to detect MTBC members in clinical tissue samples from cattle. Specific primers and a hybridization probe were used to amplify MTBC-specific sequences in order to avoid cross-reaction with non-MTBC species. An Internal Amplification Control (IAC) was included in order to assess the presence of PCR inhibitors in the samples. The PCR was optimized to achieve maximum efficiency, and the limit of detection, limit of quantification and dynamic range of the reaction were determined. The specificity of the reaction was tested against 34 mycobacterial and non-mycobacterial species. The diagnostic sensitivity, specificity and positive and negative predictive values (PPV and NPV) of the method were assessed on 200 bovine tissue samples in relation to bacteriological culture. The dynamic range of the reaction spanned from 5 ng/reaction (10^6^ genome equivalents) to 50 fg/reaction (10 genome equivalents). The efficiency of the reaction was 102.6% and the achieved R^2^ was 0.999. The limit of detection with 95% confidence was 10 genome equivalents/reaction. No cross-reactions with non-MTBC species were observed. The diagnostic sensitivity and specificity values of the *mpb70* specific Real-Time PCR respect to culture were 94.59% (95% CI: 86.73–98.51%) and 96.03% (95% CI: 90.98–98.70%), respectively, with a PPV of 93.33% (95% CI: 85.55–97.07%) and a NPV of 96.80% (95% CI: 92.10–98.74%). The concordance of the Real-Time PCR based on *mpb70* is comparable to that of culture (K = 0.904) showing a great potential for the detection of members of the MTBC in animal tissues.

## Introduction

Bovine tuberculosis (bTB) is a chronic infectious disease caused by members of the *Mycobacterium tuberculosis* complex (MTBC), which affects certain species of mammals including cattle (1). Within this group of bacteria, *M. bovis* followed by *M. caprae* are the most frequent species in bovines. Due to its zoonotic potential and to the economic importance of cattle in the EU, this disease is subject to well-established national eradication campaigns in Member States. According to the legislation in force, i.e., 64/432/ECC, the intradermal tuberculin test is the official test in order to classify TB free herds, areas or countries, and microbiological culture is the method of confirmation of MTBC infections in bovine tissues.

The reported recovery rates for culture in general oscillate between 30 and 95% (2–5) while in a recent study using a Bayesian approach, the diagnostic sensitivity and specificity of culture was 78.1 and 99.1%, respectively (6). This variation between studies can be explained by different factors associated with the technique and the samples, which can affect the performance of the method. Firstly, the choice of tissue samples at the abattoir is a key for culture. Abnormal lymph nodes and parenchymatous organs with bTB-compatible lesions must always be included when present. If pathological lesions are not detected then, specific lymph nodes (retropharyngeal, bronchial, mediastinal, supramammary, mandibular, and mesenteric) should be taken for examination and culture. Secondly, the preservation of samples until culture by refrigeration or freezing, together with the step of chemical decontamination, is mandatory in order to decrease the risk of contamination with other microorganisms. Inadequate storage and sample treatment influence the viability of MTBC and can promote the growth of contaminating microorganisms (2). Thirdly, the type of culture media chosen to grow mycobacteria may influence the recovery rate of microbiological culture. MTBC growth can be detected either by colony formation in agar and egg-based solid media (such as Middlebrook 7H10/7H11, Stonebrink or Löwenstein-Jensen with sodium pyruvate), or fluorescence or pressure differences in liquid media (BACTEC 460 TB and MGIT 960 and VersaTREK system). In studies comparing both culture systems, the recovery rates for liquid media are higher than those reported for solid media with values of 80 to 95% and 65 to 82%, respectively (3, 5). The highest recovery rates within liquid systems are recorded for the BACTEC 460 TB system, which is no longer commercially available. In addition, there is a suspected decrease in selectivity of the MGIT 960, which in turn makes liquid media more prone to overgrowth by rapidly growing microorganisms (4, 5). Members of the MTBC are grouped within the slow growing mycobacteria due to their slow replication cycle. As a result, culture detection of MTBC is extremely slow; around 28 days for liquid media and 43 days for solid media for a positive result (2, 3).

In order to overcome the problems associated with the recovery of MTBC by culture, detection of mycobacterial DNA from animal tissue samples using PCR is being considered as an alternative or complementary test to microbiological culture. Since the early 90's, many conventional PCRs have been developed and used for the direct detection of members of the MTBC in bovine samples (7). In those studies including fresh bovine tissue samples from animals with visible and non-visible lesions (VL and NVL), the reported sensitivity and specificity values of PCR with respect to culture showed great variability, ranging from 63 to 97%, and 50 to 97%, respectively (8–10). After the introduction of Real-Time PCR for the detection of MTBC species, sensitivity values increased with respect to conventional PCR. In those studies implementing Real-Time PCR in which bovine tissue samples with VL and NVL were analyzed, diagnostic sensitivity and specificity by Real-Time PCR ranged between 74 to 100% and 97 to 100%, respectively (6, 11–14). The variability in the values between studies depends not only on the type of PCR (conventional, nested or Real-Time PCR), but also on the PCR target (single- or multiple copy) and reagents, the type and number of samples included in the studies, and the DNA isolation methods. The largest study to date assessing the diagnostic performance of Real-Time PCR for the detection of MTBC using a Bayesian approach reported a diagnostic sensitivity of 87.7% and a specificity of 97% (6).

In this study, we describe the development and validation of a Real-Time PCR based on the *mpb70* gene, which encodes for a major antigenic protein conserved in all MTBC species. In addition, we assess its diagnostic performance in fresh bovine tissue samples obtained within the Spanish national eradication campaign.

## Materials and Methods

### Real-Time PCR

#### PCR Design and Optimization

The *mpb70* gene was the target of this PCR since it encodes for a majorly expressed antigenic protein in *M. bovis*, which is conserved in all members of the MTBC. An *in silico* specificity analysis was carried out, in order to rule out any sequence homologies between other bacterial species, with the Basic Local Alignment Tool (BLAST) from the NCBI, using the *mpb70* CDS from *M. bovis* AF2122/97 (NC_002945.4). The *mpb70* sequence was then used to obtain *mpb70* homologs from the available MTBC genomic sequences deposited in the genbank (NCBI): *M. tuberculosis* H37Rv (NC_000962.3), *M. africanum* strain 25 (CP010334.1), *M. caprae* Allgeau (CP016401.1), *M. microti* strain 12 (CP010333.1), *M. mungi* strain BM22813 (LXTB01000090.1), *M. orygis* strain 112400015 (APKD01000057.1) and *M. canetti* CIPT 140010059 (NC_015848.1).

Oligonucleotides targeting the *mpb70* gene, specific for members of the MTBC, were designed to target a 133bp conserved amplicon with Oligo primer analysis software 6.0 (Molecular Insights, West Cascade, CO, USA): *mpb70*-forward: 5′-CTCAATCCGCAAGTAAACC-3′, *mpb70*-reverse: 5′-TCAGCAGTGACGAATTGG-3′ (15), and *mpb70*-probe: 5′- FAM-CTCAACAGCGGTCAGTACACGGT-BHQ1-3′. The amplicon sequences were obtained and aligned against the available MTBC sequences, as well as with the closest similarities obtained in the *in silico* specificity analysis (e.g., *M. kansasii, M. indicus pranii*, or *M. marinum*).

The Real-Time PCRs were carried out using the QuantiFast® Pathogen PCR + IC Kit (QIAGEN, Hilden, Germany). This kit includes an Internal Amplification Control (IAC), as well as specific reagents and primers/probes required for its amplification. It employs MAX™NHS Ester as a reporter dye. Different primer/probe concentrations and extension temperatures were tested in order to achieve maximum replication efficiency.

*M. tuberculosis* H37Rv DNA was used for the generation of the standard curve and positive controls. Ultra-pure distilled water was used as negative controls. This strain was grown in Löwenstein-Jensen slants in the BSL3 facilities at VISAVET Health Surveillance Center. A loop full of colonies was collected and heat inactivated (100°C) in 200 μl of ultra-pure distilled water during 15 min.

The efficiency and dynamic range of the reaction were assessed in triplicates using a standard curve prepared from a stock of 10 ng/μl of *M. tuberculosis* H37Rv genomic DNA, 10-fold serially diluted to a range of 1 ng/μl to 0.1 fg/μl. DNA concentration and quality of the DNA solution were measured in ten replicates using a nano-drop spectrophotometer (ThermoFisher, Waltham, MA, USA). The dynamic range of the reaction was established as the range of standard curve concentrations at which the coefficient of linearity was >0.997 and the cycle separation between the 10-fold dilutions was close or equal to 3.32 cycles. The limit of quantification was established as the lowest concentration point of the dynamic range of the reaction.

The optimized setup with a final 25 μl volume per reaction, including the Internal Amplification Control (IAC) was: 5 μl of 5x Quantifast Pathogen Master Mix, 2.5 μl of 10x IAC assay, 2.5 μl of 10x Internal Control DNA, 2 μl of 10 pmol/μl *mpb70*-Forward primer, 2 μl of 10 pmol/μl *mpb70*-Reverse primer, 0.75 μl of 10 pmol/μl *mpb70*-probe, 5.25 μl of ultrapure sterile distilled water (Sigma-Aldrich, St. Louis, MO, USA), and 5 μl of DNA sample. Primers and probe were obtained from Eurofins Genomics (Ebersberg, Germany).

All PCR reactions were carried out in a CFX96 Touch™ Real-Time PCR Detection System (Bio-Rad, Hercules, CA, USA) according to the following optimized cycling conditions; 95°C for 5 min followed by 45 2-step cycles of 95°C for 15 s and 60°C for 30 s, with data acquisition at this step.

### Analytical Specificity and Sensitivity

The inclusivity of the PCR was tested against seven species of the *M. tuberculosis* complex: *M. tuberculosis, M. africanum, M. bovis, M. bovis* BCG, *M. caprae, M. microti*, and *M. pinnipedii*. Selectivity was assessed using a panel of 69 strains from 24 Non-Tuberculous Mycobacteria (NTM) species and 10 non-mycobacterial species (OM: Other Microorganisms); *M. avium* subsp. *hominissuis* (*n* = 7)*, M. avium* subsp. *avium* (*n* = 3)*, M. avium* group X (*n* = 10), *M. chitae, M. colombiense, M. europeum, M. flavescens, M. fortuitum* (*n* = 3), *M. gordonae, M. hibernae, M. intracellulare, M. kansasii* (*n* = 9), *M. marinum* (*n* = 2), *M. neoaurum, M. nonchromogenicum* (*n* = 4), *M. parascrofulaceum, M. peregrinum* (*n* = 2), *M. phlei* (*n* = 2), *M. scrofulaceum, M. seoulense, M. shimodei, M. smegmatis* (*n* = 2)*, M. terrae, M. thermoresistible, M. vaccae, Brucella mellitensis, Brucella abortus, Salmonella enterica* Sv. Typhimurium, *Serratia maucencens, Rhodococcus equi, Enterococcus hirae, Lysteria monocytogenes, Nocardia* sp., *Streptomyces* sp., and *Corynebacterium pseudotuberculosis*. DNA for these bacteria was obtained from reference strains and clinical isolates from the VISAVET Health Surveillance Center (Complutense University of Madrid).

The analytical sensitivity or limit of detection (LOD) and intra-assay repeatability were estimated using a new standard curve that was prepared from a 10-fold serially diluted stock of *M. tuberculosis* H37Rv genomic DNA ranging from 1 ng/μl to 10 fg/μl, and one 1:5 dilution thereof to a concentration of 2 fg/μl (10 fg/reaction or 2 genomic equivalents). The reaction was carried out using 20 replicates per concentration and the LOD was established as the concentration in which at least 95% of the replicates were positive. Inter-assay repeatability was assessed in 20 replicates of 10 fg/μl *M. tuberculosis* DNA in a period of 6 months.

*M. tuberculosis* H37Rv genomic equivalents were obtained from the amount of DNA used for each point of the standard curve using the equation previously described (16): [ng of DNA × 6.023 × 10^23^ molecules/mol]/[bp length of genome × 10^9^ ng/g × 660 g/mol]. The genome size recorded at the NCBI Genome entry of *M. tuberculosis* H37Rv (NC_000962.3) was used as a reference (i.e., 4.41 Mb). Genomic equivalents for each sample were obtained by extrapolating the Ct values with the quantities from the standard curve.

### Selection, Preparation and Culture of Clinical Samples

Two-hundred fresh tissue samples from cattle were randomly selected from samples processed as part of the Spanish national eradication campaign against bTB during the period 2013–2017, based on the Royal Decree 727/2011. Processing took place within the BSL3 facilities of VISAVET Health Surveillance Center. Simple randomization was carried out by assigning a random value to each sample and by sorting them by increasing order. The first 200 samples of this list were included in the study. The selected tissue samples originated from 11 out of the 17 autonomic regions of Spain. Almost half (*n* = 99) of the samples were obtained from Madrid, followed by Castile-La Mancha (*n* = 34), Aragon (*n* = 18), Extremadura (*n* = 13), Valencia (*n* = 12), Murcia (*n* = 8), La Rioja (*n* = 6), Andalusia (*n* = 3), Canary Islands (*n* = 3), Balearic Islands (*n* = 3), and Castile and Leon (*n* = 1).

From the total amount of samples, 118 came from cattle that were positive to the single intradermal tuberculin (SIT) test (bovine PPD ≥ 4 mm), whereas 63 were from SIT-negative animals (bovine PPD ≤ 2 mm) and 4 had inconclusive results (2 mm > bovine PPD < 4 mm) according to the Royal Decree 727/2011. Following the regulation in force, in regions with high prevalence of bTB, SIT inconclusive results were considered as positive. Fifteen animals showed bTB-compatible lesions during routine abattoir inspection of carcasses and were also sent for sample collection and processing. Lymph nodes (retropharyngeal, mandibular, mediastinal, bronchial, prescapular, mesenteric, hepatic, and/or supramammary) and/or organs were then collected for processing, culture and direct PCR.

Once in the laboratory, all tissue samples were visually inspected for lesions and sliced. A total of 78 samples had VL, whereas 122 had NVL. Approximately 2–2.5 g of tissue sample from the same animal were pooled and homogenized in 12 ml sterile distilled water in a Masticator (IUL, Barcelona, Spain) at max speed for up to 5 min. One ml of the homogenized sample was collected for DNA isolation, whereas the remainder of the homogenate was decontaminated with an equal volume of 0.75% (w/v) hexadecyl pyridinium chloride solution in agitation during 30 min (17). Samples were centrifuged during 30 min at 1,300–1,500 g. Pellets were collected with swabs and cultured in Löwenstein-Jensen with sodium pyruvate and Coletsos media (Difco, Spain) at 37°C for a maximum of 3 months. Culture was considered positive when isolates were identified as MTBC by conventional PCR (18) and /or DVR-spoligotyping (19).

### Tissue DNA Extraction

DNA from tissues was obtained using the DNeasy Blood & Tissue Kit (QIAGEN) with a few modifications. Briefly, one ml of the homogenized tissue sample was added in a tube containing 100 mg of 0.5 mm and 50 mg of 0.1 mm glass beads and centrifuged for 5 min at 9,000 g. The supernatant was removed from the samples and 200 μl of sterile distilled water and 180 μl of ATL Buffer were added. Samples were then lysed in a Fastprep^®;^ FP120 homogenizer (MP Biomedicals, Santa Ana, CA, USA) using 3 cycles of 40 s at a speed of 6.5 m/sec. After an overnight chemical treatment with 20 μl of proteinase K at 56°C, the mechanical lysis step was repeated. Samples were then centrifuged briefly at maximum speed and 300 μl of supernatant were transferred to a new 1.5 ml Eppendorf tube and mixed with 400 μl of a mixture of AL buffer and 96% ethanol (equal volumes). The lysate was transferred to a spin column and was processed according to the manufacturer's instructions. DNA elution was carried out using 200 μl of AE buffer.

### Diagnostic Performance

The diagnostic performance of the Real-Time PCR targeting the *mpb70* gene was assessed on 200 randomly selected tissue-extracted DNA samples. The exogenous heterologous IAC supplied with the kit was used to assess the presence or absence of inhibition phenomena. According to the manufacturer, the IAC should show Ct values of 30 ± 3. As a result, complete inhibition was defined when no IAC was amplified and partial inhibition was defined as a Ct > 33 for the IAC. If inhibition was detected, samples were diluted 5-fold and PCR was repeated. Results were compared against microbiological culture, and diagnostic sensitivity and specificity, Positive and Negative Predictive Values (PPV/NPVs) as well as Positive and Negative Likelihood Ratios (PLR and NLRs) were calculated using MedCalc 18.2.1 (MedCalc, Ostend, Belgium). Agreement between culture and Real-Time PCR results was assessed using Cohen's Unweighted Kappa in WinEpi 2.0 (20).

Samples with culture-negative and PCR-positive results were further analyzed by DVR-spoligotyping (detection of spacers) and sequencing of the 16S rRNA gene. Sequencing of a 1,030 bp fragment of the 16S rRNA gene (18) was carried out externally by STABvida (Lisbon, Portugal). The obtained sequences were analyzed using the Bioedit software version 7.1.3.0 (21). Samples that gave a positive result to either of the above two techniques were considered as true positives and were used to re-calculate the diagnostic performance of the Real-Time PCR. On the other hand, for samples with a culture-positive and PCR-negative results DNA extraction and PCR were repeated.

## Results

### *In silico* Analysis

Sequence similarity between *mpb70* homologs in members of the MTBC is 99.7–99.8% (data not shown). Even though some non-MTBC species -such as *M. kansasii, M. marinum* or *M. gilvum*- have homologous *mpt70/mpb70* sequences (22), sequence similarity with these species is limited (data not shown). Alignments of the *mpb70* amplicons with MTBC species showed 100% identity, with exception of *M. canetti* that had a T/C substitution at position 360 (*M. bovis* AF2122/97 numbering from *mpb70* CDS start). Although this substitution falls within the length of the reverse primer, it did not affect the ability of the primer to anneal to its target in *M. canetti. M. indicus pranii, M. kansasii* and *M. marinum*, had a considerably lower identity, indicating that specificity issues would be unlikely (data not shown).

### Optimization and Analytical Sensitivity and Specificity

For optimization of the PCR reaction and repeatability studies, two 10-fold diluted standard curves were prepared from a 10ng/μl stock of *M. tuberculosis* H37Rv (DNA stock concentrations with Standard Deviations or SDs of 0.35 and 0.97, respectively).

The lowest concentration of DNA detected in the standard curve by the Real-Time PCR was 10 fg/μl (50 fg/reaction or ~ 10 genomic equivalents) with all three replicates showing an amplification curve. The dynamic range of the reaction spanned from 1 ng/μl (5 ng/reaction or approx. 10^6^ genome equivalents) to 10 fg/μl (50 fg/reaction or ~ 10 genome equivalents), with an R^2^ of 0.999. The upper and lower Ct values of the dynamic range were 20.06 and 36.33, respectively. The quantification limit was set to 10 genome equivalents/ reaction. Replication efficiency was 102.60% with a slope of −3.27.

All 20 replicates with a concentration of 50 fg/reaction were positive for this PCR, whereas only 14/20 of the 10 fg/reaction aliquots were positive. The Ct values of both dilutions were, respectively, 37.07 (SD 0.98) and 38.92 (SD 1.28). Therefore, the limit of detection for this Real-Time PCR with a 95% confidence was 10 fg/μl (50 fg/reaction or 10 genomic equivalents) and the cut-off was set to a Ct < 40.

The Real-Time PCR reacted positively only against members of the MTBC and no cross-reactions were detected against any of the NTMs or non-mycobacterial species tested.

### Diagnostic Performance Compared to Microbiological Culture

Two hundred DNA samples obtained from bovine tissues were analyzed using this PCR and microbiological culture. A total of 69 samples were MTBC positive for culture, whereas 131 were negative (Table 1). Ten out of the 131 culture-negative samples showed growth of non-tuberculous mycobacteria (*n* = 4) or other microorganisms (*n* = 6) (NTM/OM). The Real-Time PCR detected 71 positive samples, with a minimum and maximum Ct values of 24.39 and 39.35, respectively and a median Ct value of 33.48. Sixty-one out of 69 positive culture samples were also positive for the Real-Time PCR targeting *mpb70*, resulting in a sensitivity relative to culture of 88.41% (95% CI: 74.3 to 94.86%). Ten of the 131 culture-negative samples were positive for the Real-Time PCR, and the specificity value was 92.37% (95% CI: 86.41 to 96.28%). Of the 10 cultures showing growth of NTM/OM, one reacted positively to the direct Real-Time PCR.

**Table 1:** Comparison of results obtained by analyzing 200 randomly selected cattle samples by microbiological culture and Real-Time PCR.

		Culture/True positives^*^			**Diagnostic performance**		
		**Result**	**+**	**−**	**Total**	**Sensitivity**	**Specificity**
Raw results	PCR	+	61	10	71	88.41% [95% CI: 78.43–94.86%]	92.37% [95% CI: 86.41–96.28%]
		–	8	121	129		
		Total	69	131	200		
Corrected results	PCR	+	70	5	75	94.59% [95% CI: 86.73% to 98.51%]	96.03% [95% CI: 90.98–98.70%]
		–	4	121	125		
		Total	74	126	200		

* *Corrected results consider as true positives: (1) those samples that were culture positive, (2) samples that were culture-negative but PCR-positive, and for which MTBC presence was demonstrated by 16S sequencing and/or spoligotyping, and (3) culture-positive and PCR-negative samples that became positive after the DNA extraction was repeated*.

The exogenous heterologous IAC used in this PCR detected complete inhibition in only 4 out of 200 samples (2%) and partial inhibition (IAC Ct > 33) in 15 out of 200 samples (7.5%). After dilution, all these samples were PCR negative. One of the completely inhibited samples and 3 of the partially inhibited samples were positive to culture.

PPVs and NPVs were 85.92% (95% CI: 76.97 to 91.76%) and 93.80% (95% CI: 88.72 to 96.67%), respectively. The positive and negative likelihood ratios were, respectively, 11.58 (95% CI: 6.34–21.14) and 0.13 (95% CI: 0.07–0.24). There was a very good correlation between culture and PCR results (Cohen's Unweighted Kappa = 0.802).

Samples with discording results between the two methods used were further analyzed. DNA isolation was repeated for the 8 culture-positive PCR-negative samples. Of these, half (*n* = 4) gave a positive result. For samples with culture-negative and PCR-positive results (*n* = 10), spoligotyping and 16S RNA sequencing were applied and the presence of MTBC DNA was confirmed in 5 of them. Of these, one presented growth by an actinomycete and 4 were negative to culture. These samples, in addition to all culture-positives, were considered to be true positives. As a result, the corrected relative sensitivity and specificity of PCR was calculated to be 94.59% (95% CI: 86.73% to 98.51%) and 96.03% (95% CI: 90.98–98.70%), respectively (Table 1). PPVs and NPVs were, then, 93.33% (95% CI: 85.55–97.07%) and 96.80 % (95% CI: 92.10–98.74%). PLRs and NLRs increased to 23.84 (95% CI: 10.08–56.37) and 0.06 (95% CI: 0.02–0.15), respectively. Correlation between culture and PCR increased to 0.904.

Among samples with VL (*n* = 78), 65 and 61 were positive to PCR and culture, respectively (Table 2). Three out of 7 culture-negative and PCR-positive samples were shown to contain MTBC DNA by sequencing or spoligotyping. Although 3 culture-positive samples were negative for this PCR, they became positive after the extraction protocol was repeated. Regarding NVL samples (n = 122), a total of 6 samples were positive to PCR whereas 116 were found to be negative. In contrast, 8 NVL samples were culture-positive and 114 samples were culture-negative. Of these culture-negative samples, 3 were positive for the Real-Time PCR, of which 2 were confirmed as true positives by sequencing or spoligotyping. On the other hand, 5 culture-positive samples were negative for the *mpb70*-specific PCR. However, one of them was positive after the repetition of the extraction protocol. After confirmation of the true positives, Cohen's Unweighted Kappa between culture and PCR for VL and NVL samples was, respectively, 0.804 and 0.685.

**Table 2:** Comparison of results obtained by analyzing 200 randomly selected veterinary samples by microbiological culture and Real-Time PCR, according to the presence or absence of anatomic lesions.

		**Culture (True positives)**		
		**+**	**−**	**Total**
VL	PCR +	58 (64)	7 (4)	65 (68)
	PCR –	3 (0)	10	10
	Total	61 (64)	17 (14)	78
NVL	PCR +	3 (6)	3 (1)	6 (7)
	PCR –	5 (4)	111	116 (115)
	Total	8 (10)	114 (112)	122

*Culture negative and PCR-positive samples were considered true positives (in brackets) after the confirmation of the presence of MTBC DNA by 16S rRNA gene sequencing and/or spoligotyping*.

### Intra and Inter-Assay Variation

The intra-assay repeatability at a concentration of 10 fg/μl showed an average Ct value of 37.07 with a standard deviation of 0.98 and a coefficient of variation of 2.63%. Inter-assay repeatability using 20 replicates from a stock of 10 fg/μl in a 6 month period showed an average Ct value of 36.70 with a SD of 1.40 and a CV of 3.82%.

## Discussion

The purpose of this study was the design, optimization, and validation of the *mpb70* Real-Time PCR for the detection of members of the *M. tuberculosis* complex directly from animal tissue samples. In addition, this study compared the diagnostic performance of this PCR and bacteriological culture using a large number of bovine tissue samples (*n* = 200) collected in the framework of the Spanish bTB eradication program.

The Real-Time PCR targeting the *mpb70* gene showed 100% of inclusivity and selectivity. Moreover, it shows good replication efficiency (102.6%), and an analytical sensitivity of at least 10 genome equivalents with 95% confidence. Furthermore, very little variation was seen at the LOD both within and between assays (CV = 2.63 and 3.82%, respectively). In addition, the linear range of the reaction spans from 5 ng/reaction (approximately 10^6^ genomic equivalents) to 50 fg/reaction (~ 10 genomic equivalents). Although this PCR was developed for the detection of MTBC, the single-copy nature of the target and the wide linear range of the reaction make this PCR a suitable candidate for absolute quantification studies of MTBC in tissues. In fact, 59 out of the 71 *mpb70* PCR-positive samples showed a Ct value within the dynamic range of the reaction (data not shown). Although the quantification was not possible due to the absence of the standard curve in all runs, the range of concentrations was estimated to be between 2.29 × 10^5^ and 63 genomic equivalents, with an average Ct value of 33.33 (~ 415 genome equivalents).

Overall, there was a good correlation between microbiological culture and PCR results in this study. Furthermore, diagnostic sensitivity and specificity values were very good when compared to microbiological culture (88.41 and 92.37%, respectively). Eight samples were negative to the direct Real-Time PCR but positive to microbiological culture. After repetition of the DNA extraction protocol, half of them became positive to the PCR. This implies that the DNA extraction protocol is very important and directly affects the sensitivity of the PCR. Several factors influence the DNA yield and quality obtained through DNA extraction protocols.

Firstly, the amount and type of processed tissue could determine the bacterial load in the sample. The extraction protocol used in this study uses a volume of sample that is 1/10 the amount of sample used for microbiological culture, which could produce a loss of sensitivity due to the decreasing amount of bacteria available for extraction. In addition, the presence or absence of lesions can affect the amount of bacteria in the sample which in turn could determine the quantity of available DNA. In this study, the four remaining culture-positive and PCR-negative samples had NVLs, of which 3 were positive to the SIT test. This suggests that the animal may have been at early stages of infection and, therefore, have low bacterial loads. On the other hand, the recovered samples after the second extraction (*n* = 4) had mostly VLs (*n* = 3).

Secondly, the type of disruption technique used can have an important effect in the DNA extraction process. Even though the protocol in this study has been optimized to obtain a high amount of DNA through two mechanical and one overnight chemical lysis steps, improvements in the extraction protocol may reduce the number of discording results. Park et al. showed that increasing the incubation time before mechanical lysis with ATL buffer up to 3 h increased the DNA yield in *M. avium* subsp. *paratuberculosis* when compared to no pre-treatment (23). On the other hand, an 8-h pre-treatment was detrimental to the amount of extracted DNA, achieving the same amount of DNA as the no pretreatment controls. The effect of reduction in the pre-treatment incubation time should be assessed in the future. Another improvement could include the use of a homogenizer instead of a masticator in the tissue homogenization step, which could release a larger amount of bacteria from tissue samples for extraction.

Furthermore, several factors associated with the extraction protocol may introduce inhibitors in the sample, such as organic compounds or excess host DNA. In order to detect the inhibition of the PCR, the reaction mix includes an exogenous heterologous IAC, with a randomly generated DNA supplied by the manufacturer. By using the IAC, 4 and 15 samples were found to be completely or partially inhibited, respectively. Of these, 1 inhibited and 3 partially inhibited samples were culture-positive, and they remained PCR-negative after a 1:5 dilution. After repeating the extraction protocol on these samples, the inhibited and one partially-inhibited sample became positive, indicating that their dilution may have caused the further dilution of the target DNA and, therefore, may have resulted in the loss of sensitivity in the PCR. Furthermore, this could imply that the inhibitor was not present in the sample and was introduced as a result of the DNA extraction procedure, or that the extraction protocol failed to remove it in the first place. Other reported PCRs also include IACs, but only a few include information regarding the presence or absence of inhibition (11–13). Although no cases of inhibition were detected in these publications, they used endogenous or exogenous homologous IACs, which may present some disadvantages with respect to exogenous heterologous IACs. For instance, the amount of endogenous IAC template (i.e., bovine β-actin gene) varies depending on the type of sample or extraction method used, which means that readouts vary between samples and there is no indication of the level of inhibition present in the sample. In addition, they can overcome inhibitory effects in the sample as they are usually in higher concentrations than the target. Exogenous homologous IAC (i.e., *M. bovis* DNA), on the other hand, are recognized by the target's primers and can, therefore, give rise to competition events that can hinder the amplification of the target DNA in low-concentrated samples, such as those close to the LOD. Exogenous heterologous IACs, such as the ones used in this study, use a consistent amount of control template, different to the target of interest, with a set amplification cycle. As a result, it allows the detection of complete or partial inhibition phenomena and minimizes competition, since the primers and the control target sequence are completely different to those of the target of interest.

Ten samples were negative to culture but positive to the Real-Time PCR. The use of spoligotyping and/or 16S rRNA sequencing on these discording samples showed the presence of MTBC DNA in 5 of them. The inability of culture to detect MTBC in these samples may be due to sample processing issues in which bacterial integrity is hampered and growth is impeded. In addition, very advanced granulomatous lesions may contain lower numbers of viable bacteria than early granulomas (24). Nevertheless, MTBC DNA can still be present in non-viable bacteria in enough quantity to be detected by PCR after purification. Although no histopathological evaluation was done on these samples, 7 of the culture-negative and PCR-positive samples were obtained from animals with VLs whereas 3 were obtained from animals with NVLs. Finally, growth of NTM/OM could be another reason for these discrepancies. In fact, 1 of the 10 tissue samples that showed growth of NTM/OM during culture was positive to this PCR, indicating that the growth of MTBC in culture could have been hampered by the growth of other NTM/OM. The detection of MTBC DNA in this sample by 16S rRNA sequencing and spoligotyping supported the analytical specificity of the *mpb70* oligonucleotides, indicating that the presence of other microorganisms in the sample will not interfere with this PCR.

When the presence of MTBC DNA was confirmed in the discording samples, these were considered as true positives. Therefore, 70 positive samples were correctly identified by PCR, increasing the diagnostic sensitivity and specificity values with respect to culture (from 88.41 to 94.59% and from 92.37 to 96.03%, respectively). PPVs and NPVs were 93.33 and 96.80%, respectively. Furthermore, the PLR was 23.84 indicating a high probability of correctly identifying a bTB-positive tissue sample. In addition, the NLR was very low (0.06), indicating a low probability of a negative result being positive.

The most commonly used genetic target for PCR detection of MTBC species is the IS*6110* transposon (25). Other targets used in the detection of MTBC members in veterinary samples through PCR include the 16S-23S rRNA Internally Transcribed Spacer or ITS (14), *hupB* (26), TbD1 (11), *rv2807* (12), and *devR* (16). The high sequence similarity between the different MTBC species and the single-copy nature of the *mpb70* gene make it also a suitable target for both detection and quantification through Real-Time PCR. Since the early 1990's, the *mpb70* gene has been used extensively for the detection of MTBC species through conventional PCR (18, 27–29). Additionally, it has been used as a target for Real-Time PCR quantification of MTBC members in infected cell culture extracts (15). However, in this study hybridization probes were added to increase specificity.

Although the diagnostic specificity of this PCR was similar (96.03 vs. 97%) to that seen for the Real-Time PCR used by Courcoul et al. targeting the IS*6110* element (6), diagnostic sensitivity was higher in this study (94.59% vs. 87.7%). When compared against a Real-Time PCR detecting the IS*6110* element based on melting curve analysis and hybridization probes (30), the *mpb70*-targeting PCR showed a better correlation with culture results and increased diagnostic sensitivity. A semi-nested Real-Time PCR targeting the IS*6110* showed very similar diagnostic sensitivity, specificity and predictive values to those obtained in this study; 100% diagnostic sensitivity, 97.7% diagnostic specificity, 96.3% PPV and 100% NPV (13). Even though the LOD is lower for this semi-nested Real-Time PCR (1.5fg vs. 50fg), the requirement of two PCR steps increases the risk of cross-contamination. In addition, a Real-Time PCR targeting the 16S-23S ITS showed a moderate diagnostic sensitivity of 73.87% (14).

It is important to consider that the diagnostic performance of this PCR in this study does not give information about the infection status of all animals included in this study, as it only compares culture and PCR on tissue samples. Based on the results of this study and previous publications, direct PCR has some advantages compared to culture for the detection of MTBC species in animal tissue samples. In the first place, PCR takes a few hours to complete in comparison to the weeks required for microbiological culture. Secondly, analytical specificity can be extremely high if the appropriate oligonucleotides are designed, limiting cross-reaction with contaminating microorganisms. This removes the requirement for a decontamination step, decreasing the hazardous conditions applied to the sample. Furthermore, it would reduce the risk of exposure to mycobacteria as it decreases the processing time of tissues with suspected MTBC infections before inactivation, the amount of time spent at BSL3 facilities and the bacterial load to which the user is exposed to. In addition, the *mpb70* PCR showed a comparable limit of detection and diagnostic sensitivity to that seen in IS*6110* PCRs. One disadvantage of the IS*6110* target over the *mpb70* is the risk of horizontal transfer of mobile elements between mycobacterial species, as has been recorded for IS*1245* and *M. kansasii* (31). Moreover, the IS*6110* is present in a variable number of copies within the genome of certain MTBC species, which limits its use in quantitative studies, unlike the *mpb70* gene, which is a single-copy gene.

The results obtained in this study open the possibility of using the direct Real-Time PCR as an alternative to microbiological culture in the short term. Although microbiological culture is still needed for bacterial isolation and molecular characterization with epidemiological purposes, PCR could decrease considerably the time needed until results are obtained, improving the decision making capacity during the eradication campaigns.

In conclusion, the Real-Time targeting the *mpb70* gene is a time-effective and efficient method for the detection of MTBC members in veterinary tissue samples, which shows improved diagnostic performance with respect to culture. In addition, it has a low detection limit of 10 genomic equivalents/reaction of MTBC species. Furthermore, being a single copy gene and having a dynamic range of 10^6^-10 genomic equivalents/reaction, it could be used for quantification studies of as little as 10 genomic equivalents.

## Author Contributions

VL-L and EL performed all experiments in this study and the *in silico* specificity analysis. EC participated in the design of the *mpb70* specific oligonucleotides used in this study. BR, EL, and LdJ designed the study. LD and LdJ are responsible for the obtaining of samples. VL-L wrote the manuscript with the invaluable insights of EC, JB, EL, LD, BR, and LdJ. BR directed and supervised the complete study.

### Conflict of Interest Statement

EC is a full-time employee of Exome Diagnostics Inc. The remaining authors declare that the research was conducted in the absence of any commercial or financial relationships that could be construed as a potential conflict of interest.
